# Raised Serum Markers of T Cell Activation and Exhaustion in Granulomatous-Lymphocytic Interstitial Lung Disease in Common Variable Immunodeficiency

**DOI:** 10.1007/s10875-022-01318-1

**Published:** 2022-07-05

**Authors:** Mai Sasaki Aanensen Fraz, Annika Elisabet Michelsen, Natasha Moe, Trond Mogens Aaløkken, Magnhild Eide Macpherson, Ingvild Nordøy, Pål Aukrust, Eli Taraldsrud, Are Martin Holm, Thor Ueland, Silje Fjellgård Jørgensen, Børre Fevang

**Affiliations:** 1 Section of Clinical Immunology and Infectious Diseases, Oslo University Hospital, Rikshospitalet, Oslo, Norway; 2 Centre for Rare Diseases, Oslo University Hospital, Oslo, Norway; 3 Research Institute of Internal Medicine, Oslo University Hospital, Rikshospitalet, Oslo, Norway; 4 Institute of Clinical Medicine, University of Oslo, Oslo, Norway; 5 Department of Radiology and Nuclear Medicine, Oslo University Hospital, Rikshospitalet, Oslo, Norway; 6 Department of Infectious Diseases, Oslo University Hospital Ullevål, Oslo, Norway; 7 Faculty of Health Sciences, K.G. Jebsen TREC, University of Tromsø, Tromsø, Norway; 8 Department of Immunology, Oslo University Hospital, Oslo, Norway; 9 Department of Pulmonary Medicine, Oslo University Hospital, Oslo, Norway

**Keywords:** Common variable immunodeficiency, interstitial lung disease, granulomatous-lymphocytic interstitial lung disease, CVID, GLILD, ILD

## Abstract

**Purpose:**

About 20–30% of patients with common variable immunodeficiency (CVID) develop granulomatous-lymphocytic interstitial lung disease (GLILD) as one of several non-infectious complications to their immunodeficiency. The purpose of this study was to identify biomarkers that could distinguish GLILD from other non-infectious complications in CVID.

**Methods:**

We analyzed serum biomarkers related to inflammation, pulmonary epithelium injury, fibrogenesis, and extracellular matrix (ECM) remodeling, and compared three subgroups of CVID: GLILD patients (*n* = 16), patients with other non-infectious complications (*n* = 37), and patients with infections only (*n* = 20).

**Results:**

We found that GLILD patients had higher levels of sCD25, sTIM-3, IFN-γ, and TNF, reflecting T cell activation and exhaustion, compared to both CVID patients with other inflammatory complications and CVID with infections only. GLILD patients also had higher levels of SP-D and CC16, proteins related to pulmonary epithelium injury, as well as the ECM remodeling marker MMP-7, than patients with other non-infectious complications.

**Conclusion:**

GLILD patients have elevated serum markers of T cell activation and exhaustion, pulmonary epithelium injury, and ECM remodeling, pointing to potentially important pathways in GLILD pathogenesis, novel targets for therapy, and promising biomarkers for clinical evaluation of these patients.

**Supplementary Information:**

The online version contains supplementary material available at 10.1007/s10875-022-01318-1.

## Introduction

Common variable immunodeficiency (CVID) is the most common symptomatic primary immunodeficiency in adults with a prevalence of 1:25,000–1:50,000 [1]. The patients are characterized by decreased levels of IgG and IgA with or without low IgM levels, with poor antibody response to vaccines (and/or low switched memory cells), and should have either increased susceptibility to infection, autoimmune manifestations, granulomatous disease, and/or unexplained polyclonal lymphoproliferation [2]. Up to 70% of CVID patients develop non-infectious inflammatory/immune-mediated complications, such as autoimmune cytopenia, interstitial lung disease (ILD), enteropathy, and liver disease [3]. CVID is a heterogeneous disorder where monogenic defects are found in ~ 10%, implying that several mechanisms of immune dysregulation can lead to these non-infectious complications [4]. It is unclear to which degree the individual clinical inflammatory complications have a shared pathogenesis, and various cells such as B cells, T cells, monocytes/macrophages, and type 3 innate lymphoid cells seem to be involved [5–9].

Approximately 20–30% of CVID patients develop ILD, which is associated with increased morbidity and mortality [10]. Pulmonary histological abnormalities in these patients reflect diversity and overlap, and include patterns of pulmonary lymphoid hyperplasia such as follicular bronchiolitis, lymphocytic interstitial pneumonitis, and nodular lymphoid hyperplasia as well as granulomas, organizing pneumonia, and fibrosis [10–13]. The term “granulomatous-lymphocytic interstitial lung disease” (GLILD) was proposed by Bates et al. to describe ILD in CVID with lymphocytic infiltrates and/or granulomas [10]. However, it is not clear if different entities of CVID-related ILD, also within the term GLILD, represent different pathological processes or variations within a disease spectrum. GLILD can often be distinguished radiologically from other ILDs by CT findings, and is characterized by reticulation, bronchial wall thickening, pulmonary nodules, and ground glass opacities [14–16].

The pathogenesis of GLILD is poorly understood. Small case series of immunohistochemistry performed on lung biopsies in GLILD have shown lymphocytic infiltrates with the presence of T cells and variable findings of B cell follicles within the infiltrates [11, 17, 18]. Maglione et al. found increased serum levels of B-cell activating factor (BAFF) in CVID patients with a progressive course of ILD compared to stable GLILD, suggesting that a BAFF-mediated resistance to apoptosis drives pulmonary B cell hyperplasia within the lungs [6, 18]. Friedmann et al. found that bronchoalveolar lavage (BAL) fluid of patients with CVID-related ILD had lower numbers of regulatory T cells, higher numbers of T follicular helper (T_FH_)-like memory cells skewed toward Th1 cells, and a larger fraction of B cells, mostly the inflammatory CD21_low_ B cell subtype, as compared to patients with sarcoidosis [19].

In idiopathic pulmonary fibrosis and connective tissue disease related ILD, biomarkers such as surfactant protein D (SP-D), Club Cell protein 16 (CC16), and matrix metalloproteinase 7 (MMP-7) have been shown to work as diagnostic and prognostic biomarkers [20–23]. SP-D and CC16 appear to reflect airway epithelium injury, and MMP-7 is a marker of extracellular matrix (ECM) remodeling and fibrosis [24–26]. To the best of our knowledge, these markers, and other markers of ECM remodeling and growth factors with relevance for fibrogenesis, have not previously been examined in GLILD.

CVID patients with an inflammatory phenotype often develop several non-infectious complications. In this study, we aimed to identify biomarkers that could distinguish GLILD, or reflect the pathogenesis of GLILD distinctively, in a population of CVID patients where all have non-infectious complications. Accordingly, we designed this study by dividing the cohort of CVID patients with non-infectious complications in those with and those without GLILD, and kept CVID with infections only in a separate group. We analyzed selected soluble serum markers of inflammation, immune homeostasis, pulmonary epithelium injury, fibrogenesis, and ECM remodeling (see Table 1 for overview) based on previous studies of ILD, CVID, and various inflammatory disorders, and aimed to distinguish the serum profile of GLILD from other non-infectious complications in CVID. This could highlight novel aspects of GLILD pathogenesis and identify new therapeutic approaches and clinically useful biomarkers.

**Table 1:** Abbreviations and descriptions of the analyzed biomarkers

Biomarker	Description and functional aspects
Central inflammatory cytokines	
BAFF	B cell activating factor, belongs to the TNF family, mediates peripheral B cell survival
IFN-γ	Interferon gamma, central in Th1 immune response and macrophage activation
IL-6	Inflammatory interleukin, stimulates acute response, hematopoiesis and immune reactions
TNF	Tumor necrosis factor, inflammatory cytokine with T cells and macrophages as sources
Leukocyte markers	
MPO	Myeloperoxidase of neutrophils, contributor to oxygen-dependent microbicidal activity
sBCMA	Soluble B cell maturation antigen, a TNF superfamily receptor activated by BAFF/APRIL
sCD14	Myeloid differentiation marker, primarily on monocytes/macrophages, released by activation
sCD25	Soluble IL-2Rα chain (sIL-2Rα), shedded in T cell activation
sCD163	Scavenging receptor of monocytes and macrophages, shedded in macrophage activation
sTIM-3	T cell Ig and mucin domain-containing protein 3, marker of T cell activation/exhaustion
Pulmonary epithelial cell injury markers	
CC16	Club cell protein 16, pulmonary protein with anti-inflammatory/antioxidant functions
PARC	Pulmonary and activation-regulated chemokine (CCL18), Th2 associated chemokine
S100A8/A9	Heterodimer with a role in cytoskeleton and leukocyte recruitment
SP-D	Surfactant protein D, neutralizing pulmonary lipoprotein complex, limits inflammation
ECM remodeling markers	
Cathepsin S	Lysosomal protease, regulates inflammation by processing cytokines and defense proteins
GDF-15	Growth/differentiation factor-15, stress-responsive cytokine expressed in multiple cells
MMP-7	Matrix metalloproteinase 7 (matrilysin), marker of ECM remodeling
MMP-9	Matrix metalloproteinase 9, directly degrades ECM proteins and regulates tissue remodeling
Periostin	ECM protein active in tissue injury, inflammation, fibrosis and tumor progression
TIMP-1	Tissue inhibitor of metalloproteinase 1, an inhibitor of MMP activity
YKL-40	(Chitinase-3-like protein 1), marker of inflammation, tissue injury and ECM remodeling
Chemokines	
Eotaxin	(CCL11), eosinophil chemoattractant cytokine
IL-8	Chemoattractant interleukin, attracts and activates neutrophils
Endothelial activation markers	
Angp2	Angiopoietin 2, proangiogenic and pro-inflammatory cytokine
PAI-1	Plasminogen activator inhibitor 1, regulates and inhibits the fibrinolytic system
PECAM-1	Platelet endothelial cell adhesion molecule-1, a vascular cell adhesion and signaling molecule
VEGF	Vascular endothelial growth factor, induces angiogenesis
vWF	Von Willebrand factor, glycoprotein essential in hemostasis and endothelial cell activation

## Materials and Methods

### Ethics

The Regional Committee for Medical and Research Ethics approved the study protocol (REK no 2012/521 and 33,256). All study participants signed a written, informed consent. The work described has been carried out in accordance with the Declaration of Helsinki.

### Patients and Subgroups

CVID patients were recruited from the Section of Clinical Immunology and Infectious Diseases, Oslo University Hospital, Rikshospitalet, Oslo, Norway. CVID was defined according to the European Society of Immunodeficiencies criteria [2]. The CVID patients were subdivided into three groups: “GLILD,” “other non-infectious complications” (OC), and “infections only” (IO). Patients with GLILD were diagnosed based on clinical and radiological features, and identified by review of medical records, including radiology reports. Chest high-resolution computed tomographies (HRCTs) of suspected GLILD patients were then reassessed, and the GLILD diagnosis was confirmed by two experienced chest radiologists in consensus [16]. The HRCT performed closest in time to the serum sampling was assessed quantitatively by a scoring tool described in a previous paper [16]. Patients were classified as progressive or stable as previously described [16, 18]. Non-infectious complications were defined according to Chapel’s classification of CVID phenotypes, including autoimmunity, polyclonal lymphocytic infiltration, and enteropathy [3]. We applied two modifications of the classification: (i) inclusion of biopsy-proven nodular regenerative hyperplasia and (ii) enteropathy defined as biopsy-proven lymphocytic infiltrations and/or persistent diarrhea after exclusion of gastrointestinal infection. Lung biopsies (trans-bronchial) were available from two GLILD-patients. All patients except two had been screened by a whole genome sequencing panel for primary immunodeficiencies.

### Blood Sampling and Storage

The serum of patients was sampled between 2006 and 2013, collected in sterile tubes, allowed to coagulate at room temperature, centrifuged at 1500* g* for 10 min, and stored at − 80 °C. Serum from healthy controls (HCs) was collected between 2010 and 2015 and stored likewise. None of the sample tubes had been thawed before. At the time of sampling, none of the patients had ongoing acute infection or immune modulating medical treatment (for corticosteroids, a daily dose ≤ 5 mg prednisolone was not defined as immune modulating).

### Serum Analyses

Serum levels of Angiopoietin 2, BAFF, Cathepsin S, CC16, GDF-15, MMP-7, MMP-9, MPO, PARC, PAI-1, PECAM-1, periostin, TIMP-1, S100A8/A9, sBCMA, sCD14, sCD163, sCD25, SP-D, sTIM-3, VEGF, vWF, and YKL-40 were measured by enzyme immunoassays using commercially available antibodies (R&D Systems, Minneapolis, MN, except for vWF from Dako, Agilent, Santa Clara, CA) in a 384 format using a combination of a SELMA pipetting robot (Analytik Jena AG, Jena, Germany) and a BioTek dispenser/washer (BioTek Instruments, Winooski, Vt). Detection of eotaxin, IFN-γ, IL-6, IL-8, and TNF was performed by a MSD U-PLEX assay kit (Meso Scale Discovery, Rockville, MD). Intra- and inter-assay CVs were < 10%. See Table 1 for abbreviations.

### B and T Cell Phenotyping

B and T cell counts and fractions of lymphocyte subsets were measured by flow cytometry in EDTA-anticoagulated blood sampled and analyzed within 14 months of the time point of serum sampling, at the Department of Immunology, Oslo University Hospital, Rikshospitalet. None of the patients received immune modulating medical treatment at the time of B/T cell phenotyping. Please see supplementary material for details, including Supplemental Figure S2.

### Statistical Analyses

The primary aim of the study was to compare levels of serum markers in GLILD versus OC. Additionally, we wanted to compare the GLILD and the IO group. The CVID cohort as a whole was also compared to HCs in a separate analysis.

When examining the differences in biomarker levels between the three groups of CVID patients, our pre-defined statistical approach was to first compare these groups with the Kruskal–Wallis test for multiple group comparisons of continuous values. If the Kruskal–Wallis test yielded a *p* value < 0.05, differences between subject groups were assessed using Dunn’s multiple comparisons test, adjusted by Bonferroni for two comparisons (GLILD vs OC and GLILD vs IO). Mann–Whitney testing was used for two-group comparisons. Categorical values were compared using the chi-square test. Spearman rank correlation test was used for correlation analyses. Stepwise forward logistic regression analysis with GLILD as dependent factor was performed on log-transformed marker values as independent predictors. Calculations were performed in SPSS (version 26, IBM, NY). *p*-values < 0.05 were considered to be statistically significant.

## Results

### Patient Characteristics

**Fig. 1:**
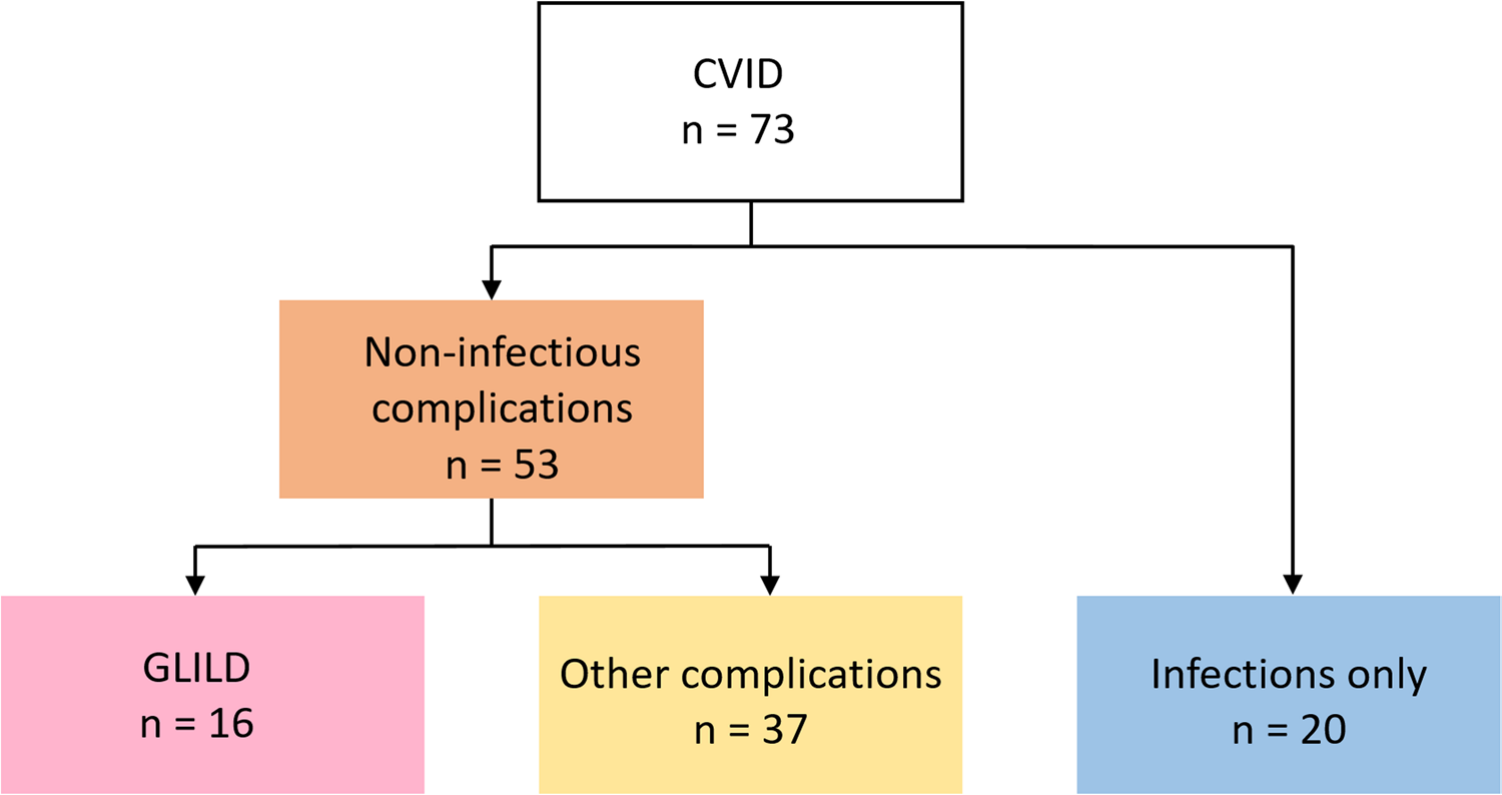
Flow chart of the CVID subgroups

The study cohort included 73 CVID patients. In total, 16 CVID patients (22%) were determined to have GLILD, 37 CVID patients (51%) had OC, and 20 CVID patients (27%) had IO (Fig. 1). These three CVID subgroups were similar in age, gender, BMI, Ig substitution form, and presence of bronchiectasis (Table 2). The distribution of complications other than GLILD was similar in the OC group and the GLILD group, except for a higher frequency of a previous history of autoimmune cytopenia in the GLILD group (38% in the GLILD group vs 8% in the OC group, *p* = 0.009). None of the patients had an ongoing episode of immune-driven cytopenia at the time of blood sampling.

**Table 2:** Characteristics of the study population

	GLILD (*n* = 16)	Other complications (*n* = 37)	Infections only (*n* = 20)	*p* value^a^	
Age (years)	42.3 (29.4)	41.2 (15.8)	42.7 (34.4)	0.993	
Female, *n* (%)	11 (69)	17 (46)	12 (60)	0.266	
Monogenic defect, *n* (%)	3 (19)	3 (8)	3 (15)	0.509	
History of smoking, *n* (%)	2 (13)	6 (17)	1 (5)	0.460	
BMI	22.6 (5.4)	26.0 (6.2)	24.2 (4.7)	0.068	
Bronchiectasis, *n* (%)	10 (63)	15 (41)	9 (45)	0.334	
Immunoglobulin administration form					
IVIG, *n* (%)	4 (25)	9 (24)	4 (20)	0.595	
SCIG, *n* (%)	12 (75)	28 (76)	15 (75)		
Ig therapy not yet started, *n* (%)	0	0	1 (5)		
Non-infectious complications other than GLILD					***p* value**^**b**^
Lymphadenopathy, *n* (%)	8 (50)	15 (41)		0.524	
Splenomegaly, *n* (%)	9 (56)	21 (57)		0.973	
Liver disease, *n* (%)	1 (6)	6 (16)		0.325	
Enteropathy, *n* (%)	3 (19)	12 (32)		0.310	
Autoimmune cytopenia, *n* (%)	6 (38)	3 (8)		**0.009**	
Granulomas in other tissue, *n* (%)	3 (19)	2 (5)		0.127	
Other autoimmune disease, *n* (%)	2 (13)	7 (19)		0.568	
Laboratory values^c^					
				***p* value**^**a**^	Normal range
CD3 + cells (× 10^6^/L)	1079 (828)	1167 (1081)	1251 (583)	0.542	800-2400
CD4 + cells (× 10^6^/L)	606 (430)	614 (328)	669 (385)	0.823	500-1400
CD8 + cells (× 10^6^/L)	292 (647)	467 (623)	575 (480)	0.420	200-1000
% T_reg_	2.0 (1.1)	3.9 (2.6)	5.1 (3.4)	**<0.001**	2.5-5.8
% follicular CD4 + memory T cells	20.0 (16.5)	21.0 (12.0)	16.5 (13.7)	0.324	8.3-20.5
CD19 + cells (× 10^6^/L)	140 (198)	131 (183)	180 (157)	0.288	100-500
% class switched memory B cells	0.2 (0.5)	0.6 (1.1)	2.1 (1.4)	**<0.001**	4.3-23.0
% transitional B cells	5.5 (12.4)	4.1 (8.5)	2.7 (6.6)	0.178	0.6-4.6
% CD21^low^ B cell	18.0 (29.0)	6.4 (16.0)	8.0 (13.2)	**0.022**	1.2-9.4
IgG (g/L)	8.1 (3.1)	6.7 (4.8)	7.1 (2.3)	0.570	6.1-14.9
IgM < 0.4 g/L, *n* (%)	12 (75)	29 (78)	14 (70)	0.782	0.7-4.3
IgA < 0.1 g/L, *n* (%)	11 (69)	33 (89)	15 (75)	0.164	0.4-2.1

Continuous data are presented as medians (interquartile range), and categorical data as frequencies (%). *p* values in bold type are statistically significant

*IVIG* intravenous immunoglobulins, *SCIG* subcutaneous immunoglobulins

^a^Calculated by the Kruskal–Wallis test for multiple group comparisons for continuous values, and by the chi-square test for categorical values

^b^Calculated by the Mann–Whitney test between GLILD and OC

^c^Missing values: five patients for absolute cell counts, seven for B cell subsets and 12 for T cell subsets

The GLILD group had one patient with a CTLA4 haploinsufficiency, one with a STAT3 *gain of function* mutation and one with a variant of uncertain significance (VUS) in STAT3. In the OC group, there were three patients with likely pathogenic genetic variants (one heterozygote variant in NFKB1, one heterozygote deletion in IKZF1, and a de novo VUS in IRF2BP2). The IO group included three patients with likely pathogenic genetic variants (one with a heterozygote variant in CTLA4, one in IKZF1, and one in NFKB2).

### Ten Markers Distinguish the CVID Subgroups: GLILD, OC, and IO

**Fig. 2:**
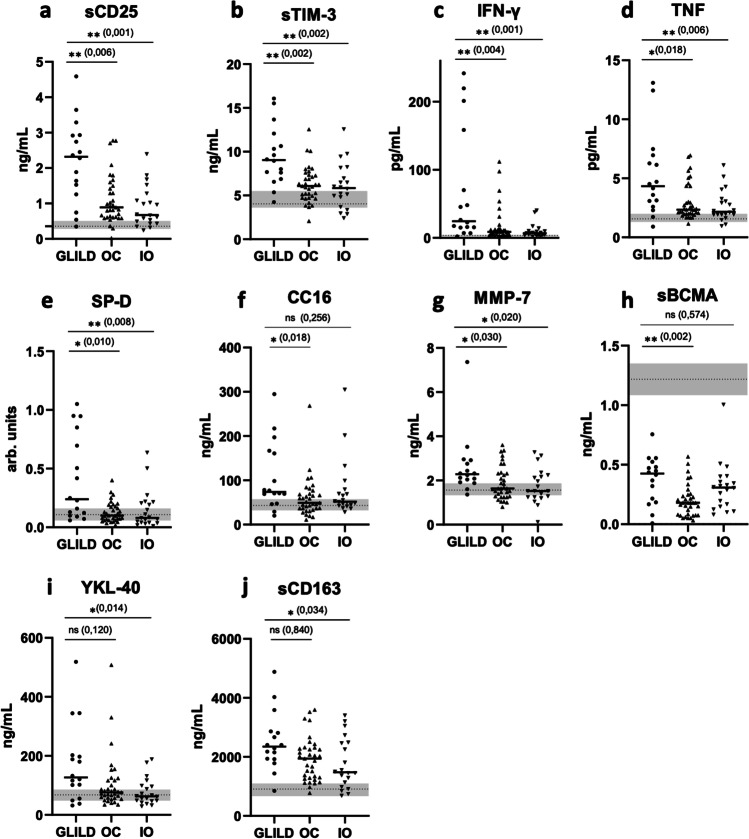
Serum markers of ten biomarkers (**a** sCD25, **b** sTIM-3, **c** IFN-γ, **d** TNF, **e** SP-D, **f** CC16, **g** MMP-7,** h** sBCMA, **i **YKL-40 and **j **sCD163) associated with GLILD in a CVID population, selected by initial Kruskal Wallis testing. *p* values over the diagrams are calculated by Dunn’s multiple comparisons test (Bonferroni adjusted) for GLILD vs OC, and for GLILD vs IO. The IQRs of HCs are marked as shaded areas with dotted line at median

The three groups, GLILD, OC, and IO, were first tested by Kruskal–Wallis test for the 28 biomarkers given in Table 1. Ten markers were significantly different between the three groups: IFN-γ (*p* = 0.001), TNF (*p* = 0.007), sCD25 (*p* = 0.001) sTIM-3 (*p* = 0.001), sBCMA (*p* = 0.002), sCD163 (*p* = 0.044), SP-D (*p* = 0.007), CC16 (*p* = 0.034), MMP-7 (*p* = 0.020), and YKL-40 (*p* = 0.025) (Supplemental table S1). The levels of these ten markers were then, as predefined, selected for further comparisons between GLILD and OC, and also between GLILD and IO (Fig. 2).

### Increased Levels of T Cell Activation Markers in GLILD

Four of the ten selected markers could be related to T cell activation: sCD25, sTIM-3 (a marker of T cell activation and exhaustion), INF-γ, and TNF (both proposed markers of Th1 cell activation). We compared their levels in GLILD vs OC and GLILD vs IO, and found that the GLILD group had significantly higher levels of all four markers compared to OC as well as IO patients (Fig. 2a–d).

### Elevated Pulmonary Epithelial Cell Injury and ECM Remodeling Markers in GLILD

Four of the selected markers could be related to pulmonary epithelial cell injury (SP-D and CC16) or ECM remodeling (MMP-7 and YKL-40), and were further explored between GLILD and OC, and GLILD and IO. We found significantly higher levels of SP-D, CC16, and MMP-7 in the GLILD group compared to the OC group (Fig. 2e–g). SP-D, MMP-7, and YKL-40 were higher in the GLILD group compared to the IO group, but not CC16 (Fig. 2e–g and i).

### Higher Levels of sBCMA in GLILD

Serum levels of BAFF that has previously been suggested as a marker of GLILD progression did in the present study not come out significantly different in initial comparisons between the three CVID groups (Supplemental Table S1). In contrast, the levels of its soluble (s) receptor form, sBCMA, were significantly different between the three groups, and were compared between GLILD and OC, and GLILD and IO. We found markedly higher serum levels of sBCMA in the GLILD group compared to OC, but not compared to the IO group (Fig. 2h).

### Higher Levels of sCD163 in GLILD Compared to IO

The monocyte/macrophage activation marker sCD163 was also differently regulated between the three groups, but we did not find a significant difference in serum levels of sCD163 between GLILD and OC, although the levels were significantly higher in the GLILD group compared to the IO group (Fig. 2j).

### IFN-γ, sBCMA, and SP-D Were the Strongest Predictors of GLILD

We performed stepwise forward logistic regression of the eight biomarkers that were significantly higher in GLILD than OC, and found IFN-γ, sBCMA, and SP-D to be the strongest predictors of GLILD (*p* = 0.017, 0.016, and 0.039, respectively). GLILD patients had more frequently a history of autoimmune cytopenia. However, if we added AI cytopenia as a covariate in the logistic regression model, IFNγ, BCMA, and SP-D still come out as the strongest predictors of GLILD (data not shown).

### Most Markers Upregulated in GLILD Were Stable Over Time

We had serial samples in 25 of the 73 CVID patients (eight from the GLILD group, 12 from the OC group, and five from the IO group), with a time interval between first and second sampling ranging from 0.5 to 6.4 years. None in the GLILD or the OC group had immune modulating treatment between the samplings that could have affected the results, and none of the GLILD patients had significant disease progression between the first and second sampling. The levels of sCD25, sTIM-3, INF-γ, TNF, SP-D, CC16, and MMP-7, which all were upregulated in GLILD, were stable over time (Supplemental Figure S1). In contrast, sBCMA had a significant decrease from the first to the second time point of sampling (Supplemental figure S1).

### GLILD Patients Had Distinct Characteristics of Lymphocyte Subsets

Absolute counts of CD4^+^, CD8^+^, and CD19^+^ cells did not differ significantly between the three CVID subgroups (Table 2). The fraction of regulatory T cells (T_reg_) was however significantly lower in the GLILD group compared to both the OC and the IO group (*p* = 0.004 and *p* < 0.001 respectively), and the fraction of class-switched memory B cells was also lower in the GLILD group compared to OC and IO (*p* = 0.038 and *p* < 0.001 respectively). For CD21^low^ B cells, the fraction in the GLILD group was significantly higher than in the OC group and in the IO group (*p* = 0.022 and 0.034 respectively). The fractions of follicular like CD4^+^ T cells were not different between the three groups (data not shown).

### Levels of SP-D Were Higher in Patients with Progressive GLILD

Four of the 16 GLILD patients had progressive GLILD, as defined by pulmonary function tests over time, and the progressive patients had significantly higher levels of SP-D than those with stable GLILD (median of 0.899 and 0.144 arb. units respectively, *p* = 0.013). We did not find any significant difference in sCD25, sTIM-3, INFγ, TNF, MMP-7, CC16, or BCMA between patients with progressive and stable disease.

### Levels of Serum Markers Did Not Correlate to Pulmonary CT Score

We could not find any significant correlations between the biomarkers specifically elevated in GLILD (sCD25, sTIM-3, IFNγ, TNF, SP-D, CC16, MMP-7, or sBCMA) and the total pulmonary CT pathology score, nor between the biomarkers and each radiological feature of GLILD.

### Several Markers Distinguish CVID from Healthy Controls

Although detecting differences in serum markers between CVID patients and HCs was not the primary aim of this study, we also observed that CVID patients overall had significantly higher levels of Cathepsin S, S100A8A9, PARC, and GDF15, as well as significantly lower levels of MMP-9, periostin, Angiopoietin 2, and PAI-1 compared to 40 sex- and age-matched healthy controls (Supplemental Table S2).

## Discussion

In the present study, we compared CVID patients with GLILD to CVID patients with other non-infectious complications to explore whether GLILD patients are characterized by a distinct profile of serum markers reflecting inflammation, pulmonary epithelial cell injury, ECM remodeling, and/or fibrogenesis. Our main findings were (i) GLILD patients had higher levels of sCD25, sTIM-3, TNF, and IFN-γ than CVID patients with OC, pointing to T cell activation and exhaustion as potentially central in GLILD pathogenesis. (ii) GLILD patients had higher levels of SP-D, CC16, and MMP-7; biomarkers of pulmonary epithelium injury; and ECM remodeling, compared to OC. (iii) Levels of BAFF were not significantly higher in our GLILD cohort, but its receptor, sBCMA, had increased levels in GLILD patients compared to the OC group. (iv) The significant markers reflecting T cell activation, airway epithelium injury, and ECM remodeling were shown to be consistent over time.

An increasing number of studies have highlighted the importance of T cell activation in the pathogenesis of the non-infectious complications of CVID [7, 8, 27, 28]. We have previously shown that sCD25 is increased in CVID compared to healthy controls, and also that sCD25 is significantly higher in CVID patients with non-infectious complications compared to infection only [29]. However, only a few studies have explored the role of T cell activation in GLILD. Berbers et al. found increased expression of Ki67 and IFN-γ in effector/memory CD4^+^ T cells as a sign of activation in patients with non-infectious complications compared to those with infections only, and pointed to more extreme findings in the GLILD patient subgroup [7]. Van Stigt et al. found elevated levels of sCD25 in a cohort of 12 patients with CVID and granulomatous disease (of which ten had GLILD/granulomas in lungs) compared to CVID patients with infection only [30]. In the present study, we extend these findings in several ways. By examining a larger CVID population than in previous reports, we found elevated levels of sCD25 in GLILD patients not only as compared to IO patients, but also compared to CVID patients with other non-infectious complications. sCD25 is released from the cell membrane of T cells as a result of activation, and has shown to be a useful biomarker in diseases such as sarcoidosis and hemophagocytic lymphohistiocytosis [31]. Our findings suggest that sCD25 could also be a relevant biomarker in GLILD.

Persistent T cell activation is often accompanied by T cell exhaustion, a dysfunctional state of activation, rendering the cell refractory to further stimuli. This can be reflected by upregulation of various check-point inhibitors like TIM-3. The soluble form of TIM-3 is a result of ADAM10-mediated shedding of its membrane-bound form, mainly on IFN-γ-producing CD4^+^ and CD8^+^ T cells [32]. Although the functional consequences of this shedding is not clear, levels of sTIM-3 have been shown to be a reliable marker of T cell activation/exhaustion in various disorders like progressive HIV, hepatitis virus C infection, and COVID-19 [32–34]. A few authors have suggested that T cell activation in CVID may be accompanied by exhaustion [8, 35], but the present study is the first to relate elevated sTIM-3 to CVID and GLILD. Although our finding of elevated sTIM-3 in GLILD is indicative of T cell exhaustion, parallel cellular studies should be performed in order to draw more firm conclusions. In relation to T cell homeostasis, we also found that fractions of T_reg_ that we previously have reported lower in CVID patients with an inflammatory phenotype were lower in the GLILD group compared to the OC group [36]. This points to a distinctive immune dysregulation in GLILD.

In line with the role of IFN-γ-producing CD4^+^ and CD8^+^ T cells as cellular sources of sTIM-3, we found elevated levels of the Th1-derived cytokine IFN-γ in GLILD patients compared to the other CVID groups. Although TNF have several cellular sources, Th1 cells are important contributors, and the raised TNF levels in GLILD patients further underscore the role of Th1 cells in GLILD. To summarize, higher levels of sCD25 and sTIM-3 combined with the Th1 response signature cytokines TNF and IFN- γ in GLILD are consistent findings suggesting that activated T cells play an important role in GLILD pathogenesis.

In the present study, we have showed that GLILD patients have higher levels of CC16 (an anti-inflammatory protein secreted by non-ciliated respiratory epithelium), SP-D (a neutralizing, anti-viral lipoprotein complex secreted by type II alveolar cells and bronchiolar epithelium [25, 37]), and MMP-7 (a protease expressed by epithelial cells, fibroblasts, and macrophages that activates microbicidal α-defensins [24]) than OC patients. In ILDs, elevated levels of CC16 and SP-D may reflect airway epithelial injury [21, 26]. Levels of CC16 are demonstrated to correlate with disease activity of ILD in systemic sclerosis, and SP-D has shown to be useful to diagnose and predict risk of exacerbation and mortality in idiopathic pulmonary fibrosis [38, 39]. SP-D in combination with MMP-7 can facilitate the identification of rheumatoid arthritis associated ILD at an early stage [40]. In GLILD, more than half of the patients have a progressive phenotype, with declining DLCO and FVC over time [16, 18]. This study suggests that CC16, SP-D, and MMP-7 could serve as biomarkers in GLILD even if their prognostic value is currently uncertain. In fact, SP-D came out as one of the strongest predictors of GLILD by stepwise regression. In line with previous reports, we found that GLILD patients had higher percentage of CD21^low^ and lower percentage of class-switched memory B cells [41, 42]. However, unlike Maglione et al., we were not able to demonstrate significantly higher levels of BAFF in the GLILD group. Instead, we did find higher levels of sBCMA in the GLILD group compared to the OC group. BCMA is shedded as sBCMA from plasma cells, and regulates plasma cell proliferation in bone marrow [43]. CVID patients overall are known to have substantially lower levels of sBCMA than healthy subjects, as confirmed in our cohort, reflecting the maturation defect of the B-cell lineage [44]. Although BCMA is mainly expressed on plasma cells, we cannot say if these or other cells are the source of sBCMA in GLILD. This issue may be subject for further studies, preferentially of bone marrow and lung biopsies of GLILD patients.

Currently, there is no consensus regarding optimal treatment of GLILD; however, rituximab combined with azathioprine or mycophenolate is often used and has shown effect in retrospective studies [6, 45]. Our results suggest that the use of treatment targeting T cells should be further explored. Although rituximab directly targets CD20^+^ cells, it also limits T cell activation by impairing B cell antigen presentation. Data on more T cell-specific therapy for GLILD are scarce. However, in a pilot study of treatment with the CTLA-4 Ig fusion protein abatacept, Warnatz et al. reported favorable results in five of eight CVID patients with ILD [46], and a case report of two GLILD patients describes successful treatment with the mTOR inhibitor sirolimus [47]. Inhibitors of the Janus kinase (JAK)/signal transducer and activator of transcription (STAT) pathway suppress intracellular signaling mediated by multiple cytokines, also afflicting T cells directly [48]. JAK inhibitors have been used to treat inflammatory conditions of some inborn errors of immunity: STAT3 and STAT1 gain-of-function mutations and type I interferonopathies, but there are as far as we know no reports of their use in CVID or GLILD.

The main strength of this study is a well-characterized CVID cohort with a substantial number of patients in all three subgroups, GLILD, OC, and IO. Another strength is that we used a simple and predefined statistical design with handpicked relevant serum markers for CVID and ILD/GLILD rather than using fixed multiplex panels. One limitation is that even though we compared GLILD patients to a CVID group of other non-infectious complications, we cannot rule out that other differences between the groups could have affected the results.

Studies in larger GLILD cohorts could clarify if the biomarkers we identified correlate with pulmonary function, treatment effect, and prognosis. The retrospective design is an important limitation of the study, as is the lack of data from bronchial lavage fluid and pulmonary biopsies from GLILD patients.

## Conclusions

Our findings suggest that T cell activation and exhaustion, pulmonary epithelium injury, and ECM remodeling are central and distinct features of GLILD pathogenesis potentially reflecting novel targets for therapy and promising biomarkers for clinical use. However, there is a need for larger prospective studies that also include pulmonary biopsy material.

## Supplementary Information

Below is the link to the electronic supplementary material.

Supplementary file1 (DOCX 266 KB)

## Data Availability

The datasets analyzed during the current study are not publicly available due to Norwegian legislation regarding general data protection regulation but are available from the corresponding author (MSAF), on reasonable request.
