# Supplementary material for: Raised Serum Markers of T Cell Activation and Exhaustion in Granulomatous-Lymphocytic Interstitial Lung Disease in Common Variable Immunodeficiency
Source: J Clin Immunol. 2022 Jul 5;42(7):1553–63. doi: 10.1007/s10875-022-01318-1 (PMC9255534; doi:10.1007/s10875-022-01318-1)
Supplement: Supplementary file 1 — Supplementary file1 (DOCX 266 KB) [file 10875_2022_1318_MOESM1_ESM.docx]

Supplementary information

Title: Raised Serum Markers of T-cell Activation and Exhaustion in Granulomatous-Lymphocytic Interstitial Lung Disease in Common Variable Immunodeficiency

Journal: Journal of Clinical Immunology

Authors: Fraz MSA^1,2^, Michelsen AE^3,4^, Moe N^5^, Aaløkken TM^4,5^, Macpherson ME^3,6^, Nordøy I^1,3^, Aukrust P^1,3,4,7^, Taraldsrud E^8^, Holm AM^4,9^, Ueland T^3,6,7^, Jørgensen SF^1,3^, Fevang B^1,2,3^

1. Section of Clinical Immunology and Infectious Diseases, Oslo University Hospital, Rikshospitalet

2. Centre for Rare Diseases, Oslo University Hospital

3. Research Institute of Internal Medicine, Oslo University Hospital Rikshospitalet

4. Institute of Clinical Medicine, University of Oslo

5. Department of Radiology and Nuclear Medicine, Oslo University Hospital, Rikshospitalet

6. Department of Infectious Diseases, Oslo University Hospital Ullevål

7. Faculty of Health Sciences, K.G. Jebsen TREC, University of Tromsø

8. Department of Immunology, Oslo University Hospital

9. Department of Pulmonary Medicine, Oslo University Hospital

Corresponding author: Mai Sasaki Aanensen Fraz

E-mail address: mai.sasaki.aanensen@gmail.com

**Supplemental fig. S1**


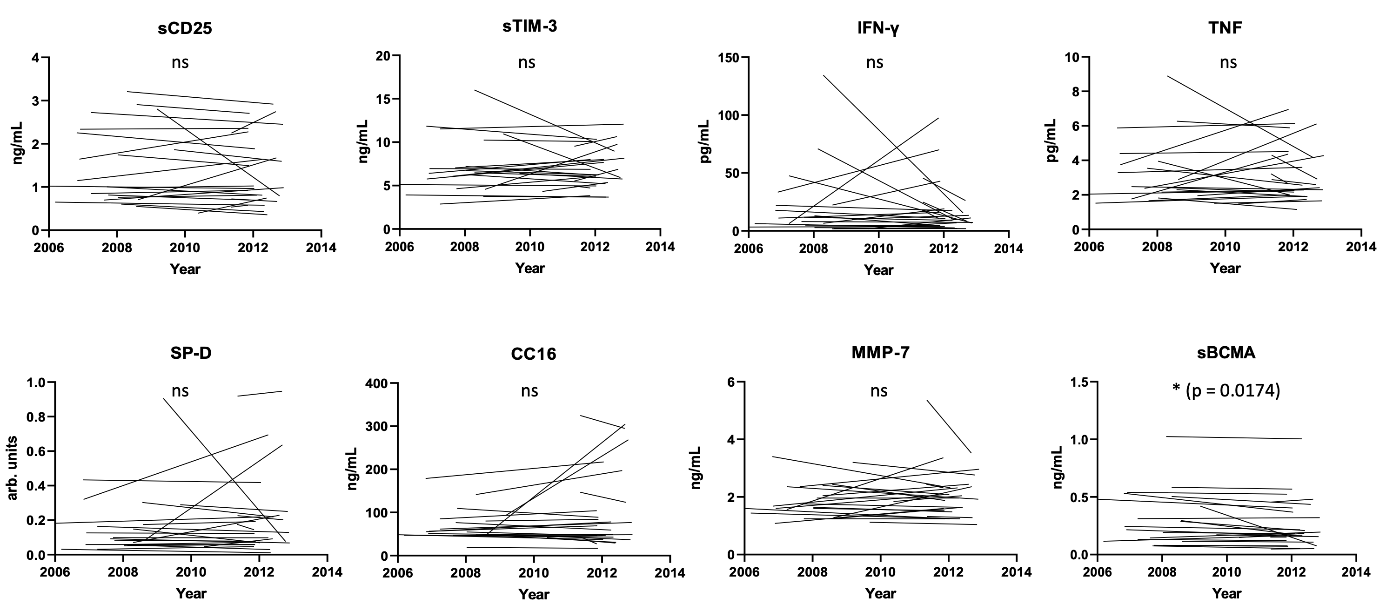


Serial samples on 25 patients (8 GLILD, 12 OC and 5 IO), of the eight markers that were elevated in GLILD compared to OC. Values of 1st and 2nd time point of sampling are compared by Wilcoxon matched pairs rank test. Time range between 1st and 2nd time point of sampling is 0.5 to 6.4 years

**Supplemental fig. S2**

Time interval from serum sampling to B/T cell phenotyping of the 60 patients with complete data of B/T cell phenotyping (median 0.00 months, range 21 months). Thirty-nine had lymphocyte phenotyping performed at the same day as serum sampling. Twenty-one patients had phenotyping performed at a different day than serum sampling: GLILD [n = 3], OC [n= 14] and IO [n= 4]

| **Supplemental table S1: Biomarker levels in the three CVID groups** | | | |  |
| --- | --- | --- | --- | --- |
| **Biomarker** | **GLILD**  **(n = 16)** | **OC**  **(n = 37)** | **IO**  **(n = 20)** | **p value^a^** |
| ***Central inflammatory cytokines*** | | | | |
| BAFF, ng/mL | 1.79 (5.45) | 1.30 (1.06) | 1.17 (1.40) | 0.357 |
| IFN-γ, pg/mL | 24.47 (121.18) | 8.69 (8.66) | 6.82 (6.07) | **0.001** |
| IL-6, pg/mL | 1.74 (2.00) | 1.60 (1.08) | 1.11 (1.41) | 0.291 |
| TNF, pg/mL | 4.33 (4.06) | 2.35 (1.68) | 2.17 (1.29) | **0.007** |
| ***Leukocyte markers*** | | | | |
| MPO, ng/mL | 440.0 (116.0) | 513.6 (361.6) | 508.8 (328.8) | 0.491 |
| sBCMA, ng/mL | 0.426 (0.321) | 0.180 (0.172) | 0.308 (0.236) | **0.002** |
| sCD14, ng/mL | 790 (172) | 736 (108) | 772 (140) | 0.120 |
| sCD25, ng/mL | 2.32 (1.61) | 0.89 (0.89) | 0.67 (0.78) | **0.001** |
| sCD163, ng/mL | 2348 (923) | 1944 (1022) | 1476 (1467) | **0.044** |
| sTIM-3, ng/mL | 9.04 (4.66) | 6.08 (2.64) | 5.84 (3.90) | **0.001** |
| ***Pulmonary epithelial cell injury markers*** | | | | |
| CC16, ng/mL | 73.8 (112.3) | 49.2 (32.8) | 51.8 (33.9) | **0.034** |
| PARC, ng/mL | 13.85 (5.05) | 14.58 (3.43) | 15.11 (4.23) | 0.809 |
| S100A8A9, ng/mL | 1494 (1089) | 1728 (2010) | 1359 (1388) | 0.427 |
| SP-D, arb. units | 0.239 (0.706) | 0.100 (0.119) | 0.081 (0.174) | **0.007** |
| ***ECM remodeling markers*** | | | | |
| Cathepsin S, ng/mL | 30.45 (9.56) | 28.36 (4.58) | 25.76 (5.66) | 0.056 |
| GDF-15, ng/mL | 0.842 (0.832) | 0.484 (0.392) | 0.488 (0.482) | 0.217 |
| MMP-7, ng/mL | 2.28 (0.93) | 1.64 (1.04) | 1.52 (0.98) | **0.020** |
| MMP-9, ng/mL | 183.6 (206.6) | 269.6 (310.4) | 256.8 (229.8) | 0.500 |
| Periostin, ng/mL | 32.40 (21.31) | 30.40 (16.00) | 32.38 (26.75) | 0.377 |
| TIMP-1, ng/mL | 553.6 (490.8) | 567.2 (300.8) | 608.0 (265.6) | 0.889 |
| YKL-40, ng/mL | 126.6 (134.1) | 76.0 (59.6) | 62.8 (51.5) | **0.025** |
| ***Chemokines*** | | | | |
| Eotaxin, pg/mL | 264.4 (184.2) | 246.7 (176.2) | 264.4 (283.7) | 0.830 |
| IL-8, pg/mL | 7.17 (5.69) | 7.73 (5.79) | 8.58 (5.47) | 0.838 |
| ***Endothelial activation markers*** | | | | |
| Angp2, ng/mL | 1.296 (0.390) | 1.152 (0.528) | 1.140 (0.498) | 0.240 |
| PAI-1, ng/mL | 1.92 (1.80) | 2.32 (1.76) | 2.50 (1.78) | 0.527 |
| PECAM-1, ng/mL | 17.00 (7.04) | 18.72 (4.10) | 18.32 (7.84) | 0.695 |
| VEGF, ng/mL | 1.096 (1.095) | 1.036 (0.952) | 1.077 (0.505) | 0.980 |
| vWF, arb. units | 0.114 (0.089) | 0.109 (0.072) | 0.132 (0.079) | 0.475 |

Biomarker levels are presented as medians (interquartile range)

^a^ calculated by Kruskal-Wallis testing between the three groups, significant values in bold type

| **Supplemental table S2: CVID versus healthy controls** | | | |
| --- | --- | --- | --- |
|  | **CVID**  **(n = 73)** | **Healthy controls**  **(n = 40)** | ***p* value**^a^ |
| Age (years) | 41.4 (24.2) | 46.0 (24.5) | 0.116 |
| Female, n (%) | 40 (55%) | 21 (53%) | 0.815 |
| ***Central inflammatory cytokines*** | | | |
| BAFF, ng/mL | 1.300 (1.285) | 0.745 (0.179) | **<0.001** |
| IFN-γ, pg/mL | 9.068 (13.818) | 3.258 (1.452) | **<0.001** |
| IL-6, pg/mL | 1.577 (1.339) | 0.346 (0.586) | **<0.001** |
| TNF, pg/mL | 2.418 (2.384) | 1.564 (0.691) | **<0.001** |
| ***Central inflammatory cytokines*** | | | |
| MPO, ng/mL | 481.6 (264.0) | 1210.4 (1524.4) | **<0.001** |
| sBCMA, ng/mL | 0.216 (0.272) | 1.218 (0.267) | **<0.001** |
| sCD14, ng/mL | 764.00 (120) | 650 (88) | **<0.001** |
| sCD25, ng/mL | 0.980 (1.184) | 0.356 (0.225) | **<0.001** |
| sCD163, ng/mL | 1944 (1160) | 905 (430) | **<0.001** |
| sTIM-3, ng/mL | 6.320 (3.200) | 4.040 (1.900) | **<0.001** |
| ***Pulmonary epithelial cell injury markers*** | | | |
| CC16, ng/mL | 58.80 (38.4) | 43.2 (25.2) | **0.013** |
| PARC, ng/mL | 14.575 (4.025) | 12.363 (3.363) | **<0.001** |
| S100A8A9, ng/mL | 1650.0 (1494.0) | 993.3 (690.0) | **<0.001** |
| SP-D, arb. units | 0.117 (0.155) | 0.108 (0.102) | 0.467 |
| ***ECM remodeling markers*** | | | |
| Cathepsin S, ng/mL | 28.567 (6.560) | 23.160 (6.160) | **<0.001** |
| GDF-15, ng/mL | 0.520 (0.5489 | 0.400 (0.247) | **0.004** |
| MMP-7, ng/mL | 1.880 (1.000) | 1.560 (0.540) | 0.051 |
| MMP-9, ng/mL | 242.4 (244.8) | 497.6 (390.2) | **<0.001** |
| Periostin, ng/mL | 31.60 (17.80) | 89.70 (26.35) | **<0.001** |
| TIMP-1, ng/mL | 569.600 (280.000) | 677.600 (176.200) | 0.061 |
| YKL-40, ng/mL | 78.4 (72.0) | 67.6 (37.7) | 0.092 |
| ***Chemokines*** | | | |
| Eotaxin, pg/mL | 255.231 (206.643) | 268.113 (155.947) | 0.601 |
| IL-8, pg/mL | 7.796 (5.574) | 9.405 (6.566) | 0.075 |
| ***Endothelial activation markers*** | | | |
| Angp2, ng/mL | 1.224 (0.492) | 1.440 (0.732) | **0.001** |
| PAI-1, ng/mL | 2.280 (1.720) | 2.72 (1.110) | **0.022** |
| PECAM-1, ng/mL | 18.32 (4.809 | 20.08 (4.18) | **0.018** |
| VEGF, ng/mL | 1.068 (0.898) | 0.524 (0.550) | **<0.001** |
| vWF, arb. units | 0.112 (0.089) | 0.110 (0.045) | 0.726 |

Age and biomarker levels in the CVID group as a whole and the HC group presented as medians (interquartile range). Gender in frequencies (%)

^a^ calculated by the Mann-Whitney test, significant values in bold type

**Supplemental methods**

***B and T cell phenotyping***

Routine B and T cell counts analysis of respective lymphocyte subsets were performed in EDTA-anticoagulated blood samples by flow cytometry at the Department of Immunology, Oslo University Hospital Rikshospitalet. Absolute counts for B-, T- and NK-cells were determined in Trucount tubes (BD, Becton Dickinson, [Franklin Lakes, New Jersey, USA](https://www.google.no/search?safe=active&q=bd+franklin+lakes,+new+jersey,+usa&stick=H4sIAAAAAAAAAOPgE-LUz9U3MCozKDZV4gAxK7ILK7S0spOt9POL0hPzMqsSSzLz81A4VhmpiSmFpYlFJalFxYtYlZJSFNKKEvOyczLzFHISs1OLdRTyUssVsoCyqZU6CqXFiTtYGQHIVyvbawAAAA&sa=X&ved=2ahUKEwisnqrHm4r2AhXE-ioKHdqdCHQQmxMoAXoECBwQAw)) using a FacsCanto II instrument and analyzed in BD FACSCanto™ Clinical Software according to the manufacturer’s instructions. Instrument settings were standardized as recommended, and daily QC run with CS&T-Beads (BD) and 7-color Setup Beads (BD). Fractions of B- and T cell subpopulations were measured using a Gallios Flow cytometer (Beckman Coulter, San Diego, CA). Briefly, washed (B-cell analysis) or unwashed (T-cell analysis) blood samples were incubated with optimally titrated antibodies (please see table below for details) for 15 minutes at room temperature and then added red cell lysis buffer /BD FACSLysing Solution). Data acquisition was performed using Kaluza Software (Beckman Coulter). For T-cells, 1 x 105 cells was acquired; for B-cells, 1x106 cells if possible. Normal ranges were established from data obtained from blood donor samples. The laboratory follows standard operational procedures and has ISO (International Standard Organization).

| Monoclonal Antibody | Fluorocrome | Clone | Vendor | RRID |
| --- | --- | --- | --- | --- |
| CD4 | FITC | RPA-T4 | BD,Pharmingen | AB_395751 |
| IgM | FITC | G20-127 | BD Pharmingen | AB_396117 |
| CD24 | PE | ML5 | BD Pharmingen | AB_395822 |
| CD3 | PerCP | SK7 | BD Biosciences | AB _2783791 |
| CD38 | PerCPCy5.5 | HIT2 | BD Pharmingen | AB_394184 |
| CD4 | APC | SK3 | BD Biosciences | AB_2868799 |
| CD25 | APC | 2A3 | BD Biosciences | AB_2819021 |
| IgD | APCH7 | IA6-2 | BD Pharmingen | AB_10645792 |
| CD45RO | FITC | UCHL1 | Beckman Coulter |  |
| CD127 | PE | R34.34 | Beckman Coulter | AB_131301 |
| CD19 | PC7 | J3-119 | Beckman Coulter | AB_10638575 |
| CD27 | PB | 1A4CD27 | Beckman Coulter |  |
| CD21 | APC | HBS | eBioscience | AB_1582217 |
| CXCR5 | PE | J252D4 | R&D | AB_2089666 |
| CD45 | PO | HI30 | Invitrogen | AB_1475776 |

The T and B cell subpopulations were defined by the following markers:

| Lymphocyte subpopulation | Markers |
| --- | --- |
| Treg (Regulatory T cell) | CD3+, CD4+, CD127-, CD25+hi in CD4+ T cells |
| Follicular like CD4+ T cells | CD3+, CD4+, CD45RO, CXCR5+ in CD4+ T cells |
| Class switched memory B cells | CD19+, IgM-, CD27+ in B cells |
| Transitional B cells | CD19+, CD38++-, IgM++ in B cells |
| CD21low B-cells | CD19+, CD38+lo, CD21-/+lo in B cells |

Frequencies of missing T and B cell data in the three CVID groups:

|  | GLILD | Other complications | Infections only |
| --- | --- | --- | --- |
| Absolute counts | 1 | 3 | 1 |
| B cell subpopulations | 1 | 6 | 1 |
| T cell subpopulations | 3 | 7 | 2 |
